# Dietary patterns and associated factors among type 2 diabetes mellitus patients attending Wolaita Sodo university comprehensive specialized hospitals, South Ethiopia

**DOI:** 10.1038/s41598-026-39574-5

**Published:** 2026-02-18

**Authors:** Amanuel Kora Moliso, Haileyesus Worku Fankasho, Wondimagegn Paulos Kumma

**Affiliations:** Department of Public Health, Wolaita Sodo University, Wolaita Sodo, Ethiopia

**Keywords:** Diseases, Endocrinology, Health care, Medical research, Risk factors

## Abstract

A controlled diet is essential for delaying disease progression and achieving optimal glycemic control among individuals with type 2 diabetes mellitus (T2DM); however, inappropriate dietary practices remain common in low-resource settings. This hospital-based cross-sectional study examined dietary patterns and associated factors among adults with T2DM attending Wolaita Sodo University Comprehensive Specialized Hospital, Southern Ethiopia. A total of 416 participants were selected using systematic random sampling. Data were collected using a structured questionnaire, including a one-week food frequency questionnaire, socio-demographic and lifestyle characteristics, nutrition knowledge, and fasting blood sugar measurements. Dietary patterns were identified using factor analysis, and multiple linear regression models were applied to assess associated factors. Two major dietary patterns were identified, jointly explaining 20.7% of the total variance in dietary intake, indicating meaningful heterogeneity in food consumption behaviors. The mixed dietary pattern, characterized by higher consumption of raw meat, boiled beef, eggs, kocho, kita, and soft drinks, accounted for 10.6% of the variance and was positively associated with being a daily laborer (β = 0.10; 95% CI 0.03, 0.18). The traditional dietary pattern explained 10.1% of the variance and was inversely associated with the presence of comorbid conditions (β = − 0.30; 95% CI–0.62, − 0.02). Distinct dietary patterns were observed among patients with T2DM, with occupational status and comorbidity burden emerging as significant determinants of dietary behavior. These findings underscore the need for culturally tailored nutrition education and dietary counseling interventions to improve diabetes management in similar resource-limited settings.

## Introduction

### Background

Type 2 diabetes mellitus (T2DM) is a major global public health challenge, accounting for the majority of diabetes cases worldwide and contributing substantially to morbidity, mortality, and health care costs. According to recent global estimates, the prevalence of diabetes has risen dramatically over the past few decades, driven by population aging, urbanization, unhealthy dietary patterns, and sedentary lifestyles. Low- and middle-income countries now bear a disproportionate share of this burden, with rapid increases in prevalence occurring alongside limited health system capacity to manage chronic non-communicable diseases effectively^1–3^.

Dietary intake plays a central role in the prevention and management of T2DM. Appropriate dietary practices are essential for maintaining glycemic control, preventing acute and chronic complications, and improving overall quality of life among people living with diabetes. Clinical and public health guidelines increasingly emphasize whole dietary patterns rather than individual nutrients, as people consume foods in combinations that may interact synergistically to influence metabolic health. Consequently, dietary pattern analysis has emerged as a valuable approach for understanding habitual dietary behaviors and their relationships with health outcomes^4–6^.

Evidence from high-income countries has consistently demonstrated that dietary patterns rich in whole grains, fruits, vegetables, legumes, and lean protein sources are associated with improved glycemic control and reduced cardio metabolic risk, whereas patterns characterized by high intakes of refined carbohydrates, saturated fats, sugary beverages, and processed foods are linked to poorer metabolic outcomes. However, dietary behaviors are strongly shaped by cultural, socioeconomic, occupational, and environmental contexts, limiting the generalizability of findings across different populations. Understanding locally specific dietary patterns is therefore critical for designing effective, culturally appropriate nutrition interventions^7,8^.

In sub-Saharan Africa, the burden of T2DM is increasing rapidly due to ongoing epidemiological and nutritional transitions. Traditional diets based on whole grains, legumes, and minimally processed foods are increasingly being replaced or supplemented by energy-dense, nutrient-poor foods. This shift is occurring alongside persistent food insecurity, poverty, and limited access to nutrition education, creating complex dietary environments for individuals with chronic diseases such as diabetes. Despite these challenges, evidence on dietary patterns and their determinants among people with T2DM in the region remains limited^9–11^.

Ethiopia is experiencing a growing burden of T2DM, particularly in urban and peri-urban areas. While national strategies emphasize lifestyle modification as a cornerstone of diabetes management, dietary counseling is often generalized and not sufficiently tailored to local food habits, cultural preferences, or socioeconomic realities. Many patients face difficulties in selecting appropriate foods, adhering to recommended meal plans, and balancing traditional dietary practices with clinical advice. In addition, nutrition knowledge, occupational status, comorbid conditions, and lifestyle factors may substantially influence dietary choices, yet these relationships are not well documented in the Ethiopian contex*t*^12,13^.

Previous studies in Ethiopia have largely focused on individual dietary components, glycemic control, or adherence to recommended diets, with limited attention given to empirically *derive* dietary patterns. Moreover, few studies have applied robust statistical methods, such as factor analysis, to identify prevailing dietary patterns among patients with T2DM and examine their associated factors. Existing evidence is further constrained by geographical concentration in selected urban centers, limiting insight into dietary behaviors in diverse settings.

Wolaita Zone, located in Southern Ethiopia, is characterized by unique cultural food practices and staple diets, including cereal- and enset-based foods. Wolaita Sodo University Comprehensive Specialized Hospital serves as a major referral center for diabetes care in the region, providing an important opportunity to investigate dietary behaviors among a heterogeneous patient population. However, to date, there is a paucity of evidence describing dietary patterns and their determinants among patients with T2DM attending this *facility?* The absence of such context-specific data hampers the development of targeted nutrition education and dietary counseling strategies tailored to the needs of patients in this setting.

Identifying prevailing dietary patterns and understanding the socio-demographic, clinical, and lifestyle factors associated with these patterns are essential for informing effective diabetes management strategies. Such evidence can support health professionals in delivering culturally appropriate dietary advice and guide policymakers in integrating nutrition-sensitive approaches into diabetes care programs.

Therefore, this study aimed to identify dietary patterns and examine their associated factors among adults with type 2 diabetes mellitus attending Wolaita Sodo University Comprehensive Specialized Hospital, Wolaita Zone*,* and Southern Ethiopia. By applying dietary pattern analysis, this study seeks to address existing evidence gaps and contribute locally relevant data to support improved nutritional management of T2DM in resource-limited settings.

### Conceptual framework of dietary pattern and associated factors among type 2 diabetes mellitus patients

**Fig. 1:**
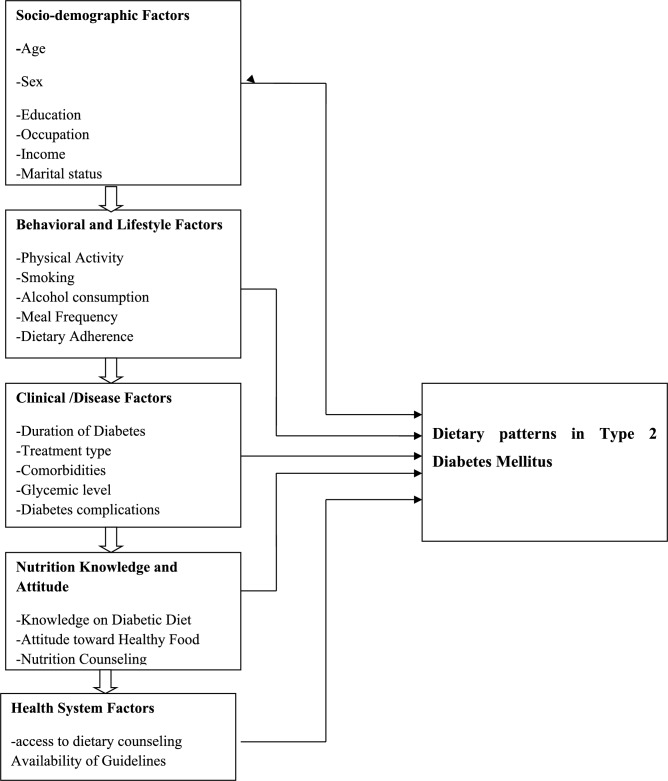
Conceptual frame work of dietary patterns and associated factors among type 2 diabetes mellitus patients^10,14–17^.

Clinical and Behavioral Factors Associated with Dietary Patterns (Fig. 1).

## Methods and materials

### Study setting

The study was conducted among adults with type 2 diabetes mellitus (T2DM) attending the diabetes follow-up clinic at Wolaita Sodo University Comprehensive Specialized Hospital (WSUCSH), located in Wolaita Sodo City, Wolaita Zone, and Southern Ethiopia. The hospital was established in 1927 by the Sudan Interior Mission and has evolved from a primary hospital to a general hospital, and subsequently to a teaching and referral hospital. In September 2021, the Ethiopian Ministry of Health officially upgraded it to a comprehensive specialized hospital.

WSUCSH serves as a major referral center for the region, providing outpatient, inpatient, and emergency services to more than 600,000 patients annually, with over 50,000 admissions per year. The hospital employs approximately 902 healthcare professionals across various disciplines, including nurses, physicians, laboratory technologists, pharmacists, anesthetists, radiologists, ophthalmic nurses, and environmental health professionals. The diabetes clinic is one of the five major outpatient departments, and on average, approximately 503 adults with T2DM attend the follow-up clinic each month.

### Study design and period

An institution-based cross-sectional study was conducted among adults with T2DM attending the diabetes follow-up clinic at WSUCSH. Data collection was carried out from March 2023 to April 2024.

### Population

#### Source population

All adults diagnosed with type 2 diabetes mellitus and registered for follow-up care at the outpatient diabetes clinic of WSUCSH constituted the source population.

#### Study population

The study population comprised systematically selected adults with T2DM who attended the diabetes follow-up clinic at WSUCSH during the data collection period**.**

### Inclusion and exclusion criteria

#### Inclusion criteria

Adults aged 18 years and above with a confirmed diagnosis of type 2 diabetes mellitus who had at least one follow-up visit prior to the data collection period were eligible for inclusion.

#### Exclusion criteria

A patient who was critically ill or required immediate medical intervention at the time of data collection was excluded from the study.

### Sample size determination

The sample size was determined using the single population proportion formula:

where n is the required sample size, Z is the standard normal value at 95% confidence level (1.96), p is the estimated proportion, and d is the margin of error (0.05).

For the primary objective, the proportion of dietary pattern prevalence (p = 44.1%) was taken from a previous study conducted in Addis Ababa among patients with T2DM. Based on this assumption, the calculated sample size was 379. After adding a 10% allowance for non-response, the final sample size was set at 416 participants.

For the secondary objective, sample size estimation was also checked using factors significantly associated with dietary patterns reported in previous studies, such as monthly income and educational status. The largest calculated sample size (n = 374) was smaller than the sample size obtained for the primary objective; therefore, the final sample size of 416 was considered adequate to address both objectives (Table 1).

**Table 1:** Sample size determination for the second specific objective based on factors associated with dietary patterns among patients with type 2 diabetes mellitus.

Relevant factor	AOR	95%CI	P = proportion	Sample size
Monthly income^18^	2.3	(1.2,4.6)	72.7%	305
Educational status^14^	2.65	(1.62,4.32)	58.2%	374

### Sampling procedure

The average monthly flow of adults with T2DM attending the diabetes follow-up clinic during the three months preceding data collection was obtained from hospital records (470, 490, and 550 patients, respectively), yielding an average monthly attendance of approximately 503 patients.

**Fig. 2:**
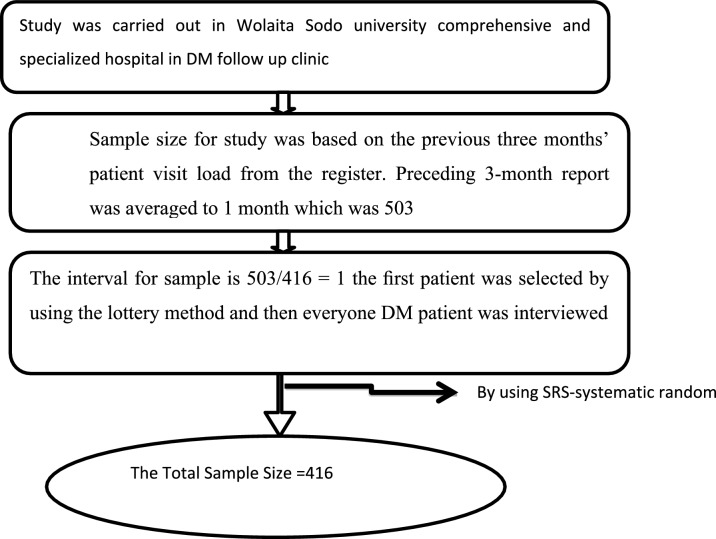
Shows Diagrammatic presentation of the sampling procedure of dietary patterns and associated factor among type 2 diabetes mellitus patients.

Systematic random sampling was employed to select study participants. The sampling interval (k) was determined by dividing the total estimated number of patients expected during the data collection period by the required sample size. Because the average monthly patient flow was comparable to the required sample size, eligible patients were recruited consecutively until the target sample size was achieved. On each clinic day, the first participant was selected using a simple random method, and all eligible patients were then approached sequentially until the required number was obtained (Fig. 2).

### Data collection tools and procedures

Data were collected using a structured interviewer-administered questionnaire consisting of socio-demographic characteristics, clinical and lifestyle factors, nutrition knowledge, and dietary intake assessment. Dietary intake was assessed using a semi-quantitative food frequency questionnaire (FFQ) containing 27 food items commonly consumed in the study area. Participants were asked to report the frequency of consumption of each food item over the previous one week. The FFQ was adapted from previous studies conducted in similar settings and contextualized to local dietary habits.

#### Questionnaire translation and quality assurance

The questionnaire was initially prepared in English and then translated into Amharic by a bilingual public health professional. A separate bilingual expert independently back-translated the Amharic version into English to ensure consistency and conceptual equivalence. Discrepancies were resolved through discussion among the research team. A pretest was conducted on 5% of the sample size at a nearby hospital not included in the study, and necessary modifications were made accordingly. Data collectors and supervisors received training prior to data collection, and daily supervision was conducted to ensure data completeness and consistency.

### Statistical analysis

Data were entered into EpiData version 4.6 and exported to SPSS version 26 for analysis. Descriptive statistics were used to summarize participant characteristics. Dietary patterns were identified using principal component analysis (PCA). The suitability of the data for PCA was assessed using the Kaiser–Meyer–Olkin (KMO) measure of sampling adequacy and Bartlett’s test of sphericity. Factors with eigenvalues greater than 1.0 were retained, and varimax rotation was applied to improve interpretability. Food items with factor loadings ≥ 0.30 were considered significant contributors to each dietary pattern. Dietary patterns were named based on the food items with the highest factor loadings.

Factor scores for each dietary pattern were calculated and used as dependent variables in multiple linear regression models to identify associated factors. Variables with a p-value < 0.25 in bivariable analysis were entered into the multivariable models. Statistical significance was declared at p < 0.05 and results were reported using standardized regression coefficients (β) with 95% confidence intervals.

### Study variables

#### Dependent variable

Dietary patterns: The dietary patterns in our study were named after the food items or groups with the highest loadings in factor analysis.

#### Independent variables

1. Socio-demographic: Age, sex, marital status, wealth index, respondents’ residence, educational status and respondent’s occupations.
2. Knowledge related factors: nutritional knowledge, doctor advice.
3. Health and health related factors: member of association, comorbidity, family history and FBS.
4. Life style factors: regular physical activity, smoking.

### Operational definitions

1. Dietary pattern: quantities, proportions, variety or combination of different foods and beverages in diets and the frequency with which they are habitually consumed.
2. Good Controlled Glycemia -Fasting blood sugar ≥ 70 mg\dl and ≤ 130 mg mg\dl.
3. Poorly controlled Glycemia -Fasting blood sugar > 130 mg\dl and ≤ 70 mg\dl.
4. High level dietary Knowledge – Study participants who had scored above mean score knowledge questions asked.
5. Low level dietary Knowledge-Study participants who had score less than mean score from knowledge questions asked.
6. Comorbidities: the presence of one or more additional disease.
7. Mixed dietary pattern: comprised animal-source foods with added sugars refined carbohydrates and consumption of carrot, dinich tibes, key sir, mucho, kik wote, kocho kita and kidney beans.
8. A traditional dietary pattern: was characterized by the consumption of carrot, Dinich tibes, key sir, mucho, kik wote, kocho kita and kidney beans.

### Ethical consideration

The ethical approval letter was obtained from Wolaita Sodo University College of Medicine and Health Science Institution of Review Board (IRB). An official supporting letter was obtained from the Department of Public health. Before data collection, informed consent was obtained from each study participant. All respondents were informed about the objective of the study and confidentiality was maintained at each step of data collection and processing. The respondents were informed that the information obtained in this study was used only for research purposes. Data collection facilitators were trained strongly on how to keep participants’ confidentiality. Also, an explanation about the rights of participants including their full right to not participate at all or withdraw at any time was provided.

All ethical procedures were conducted in accordance with the principles outlined in the Declaration of Helsinki for research involving human subjects. This ethical framework ensured the protection of participants’ rights, dignity, and well-being, and fostered mutual trust and cooperation throughout the research process.

## Result

### Socio-demographic characteristics of study participants

A total of 416 adults with type 2 diabetes mellitus participated in the study. Of these, 252 (60.5%) were male and 164 (39.5%) were female. Slightly more than half of the participants resided in urban areas (54.3%), while 45.7% were from rural settings. The mean age of participants was 47.4 years (SD ± 12.45).

Regarding marital status, 196 (47.1%) were married, 118 (28.5%) were single, 62 (15.0%) were widowed, and 37 (8.9%) were divorced. In terms of educational attainment, 102 (24.4%) had completed primary education, 137 (33.0%) had attended secondary school, 101 (24.2%) had college-level education or above, and 76 (17.9%) had no formal education.

Occupationally, 69 (16.7%) were government employees, 99 (23.9%) worked in private organizations, 109 (26.1%) were farmers, 69 (16.5%) were merchants, 59 (14.1%) were students, 25 (6.0%) were daily laborers, and 26 (6.2%) were housewives.

### Health profile of participants

Among the study participants, 153 (36.8%) reported a family history of diabetes mellitus, and 150 (36.1%) had at least one comorbid condition. Regarding diabetes management, 137 (33.0%) were using oral hypoglycemic agents, 99 (23.7%) were on insulin therapy, and only 109 (26.3%) reported using dietary modification as a primary method for glycemic control. More than half of the participants (54.3%) indicated that they had changed their dietary habits after being diagnosed with diabetes.

### Dietary knowledge of participants

Overall, 153 (36.8%) participants demonstrated a low level of dietary knowledge, while 263 (63.2%) had a high level of knowledge regarding recommended dietary practices for diabetes management.

Regarding lifestyle modification, 172 (41.1%) identified dietary modification as a means of glycemic control, 139 (33.3%) mentioned physical exercise, and 104 (24.2%) reported no knowledge of lifestyle modification strategies. Only 95 (22.7%) correctly identified carbohydrates as the food group that raises blood glucose levels most rapidly, while 112 (26.8%) were unable to identify any specific food group.

Misconceptions were common: 212 (50.7%) believed that skipping meals could help control blood glucose levels. Although 219 (52.4%) correctly identified consuming whole fruits as preferable to fruit juice, only 73 (17.5%) correctly identified that half of the plate should consist of fruits and vegetables (Table 2).

**Table 2:** Summaries detailed dietary knowledge responses od adults with type 2 diabetes mellitus attending wolaita Sodo Sodo University comprehensive specialized Hospital ,southern Ethiopia 2024.

Variables	Responses	Yes	%
Life style modification	Exercise	139	33.3
	Dietary modification	172	41.1
	I don’t know	104	24.2
Which of food groups raises blood glucose relatively faster?	Carbohydrates	95	22.7
	Fiber	130	31
	Fat	100	23.9
	Protein	27	6.5
Which of the following should you do to control your blood glucose?	Eating foods prepared only of barely	97	23.2
	To eat from different cereal	136	32.5
	To take food small or no sugar	127	30.4
	To skip breakfast and dinner	56	13.5
Which one is source of carbohydrate?	Barley, tef, bread, rice	131	31.3
	Meat,egg,milk,yoghurt	112	26.8
	Don’t know	112	26.8
Which type food group should a person with DM advised to eat most of the time?	Milk, yogurt, cheese	97	27.3
	Fat, oils ,sweets	138	33
	Vegetable and fruit	118	28.2
	Meat,fish,poultry	63	15.1
Which food helps to achieve good glycemic control? (Multiple answers are possible)	Eating fruits daily	103	24.6
	Avoiding cereals	150	34.9
	Eating fiber	103	24.6
	I don’t know	24	6
Which one is good to control blood glucose on a person with diabetes	Juices	195	46,7
	Eating fruits without juicing	219	52.4
On person with diabetes what proportion of the plate should be vegetable and fruit?	1/2	73	17.5
	1/3	63	15.1
	1/4	70	16.7
	I don’t know	208	49.6
Do you believe that skipping meal can help you control your blood glucose level?	Yes I do believe so	212	50.7
	No I don’t believe so	203	48.6
How many servings of fruits and vegetables do you think you have to eat on daily bases?	Once	119	28.5
	2–4 times	174	41.6
	I don’t now	122	29.3
Which fat do experts say is most important for people to cut down on?	Liquid oils	151	36.1
	Fats and butter	169	40.4
	Not sure	94	22.5
According to the food pyramid Which foods do you think you have to cut down a lot	Fruits and vegetables	92	22
	Milk and milk products	134	32.1
	Sweets	96	23
Which one is healthy	Butter	135	32.3
	Olive oil	182	43.5
	Don’t know	97	23.2
How many regular meals do you think you have to eat daily	One meal	107	26.7
	Two meals	157	37.6
	Three meals	104	29.4
	Don’t know	48	11.5
How many Snacks do you think you have to eat daily	One snack		
	Three Snacks	143	34.2
	Two snacks	101	24.2
	Don’t know	54	12.9

### Dietary patterns identified by principal component analysis

Principal component analysis identified two major dietary patterns that together explained 20.7% of the total variance in food consumption. The Kaiser Meyer Olkin measure and Bartlett’s test confirmed the suitability of the data for factor analysis.

The mixed dietary pattern, accounting for 10.6% of the variance, was characterized by high consumption of raw meat, kitifo (raw beef with butter and chili), boiled beef, eggs, yogurt, sugar-sweetened beverages (Coca-Cola and Sprite), tea, and baked false banana.

The traditional dietary pattern, explaining 10.1% of the variance, was characterized by higher intake of bread, potatoes, carrots, beetroot, false banana porridge (mucho), broad bean sauce, kidney beans, and local alcoholic beverages (tella) (Table 3).

**Table 3:** Factor loadings for each food item are presented in Table 3.

Dietary pattern			
Food items/groups	Traditional pattern	Mixed pattern	Uniqueness
bread	0.4149	0.2159	0.78
Macaroni	0.1869	0.3516	0.84
False banana baked	0.1714	0.4557	0.76
Potato roasted	0.472	− 0.02	0.77
Carrot	0.4172	0.0738	0.82
Keyisir(beetroot)	0.5748	0.0038	0.66
Mucho(false bannanaporridge)	0.5190	0.0169	0.73
Selata	0.2931	0.1332	0.89
Broad been sauce	0.4194	0.0808	0.81
Kidney benes	0.5431	− 0.0269	0.70
Papaya	0.2452	0.2320	0.88
Orange	0.2925	0.1979	0.87
Pineapple	0.2647	0.3411	0.81
Raw meat	− 0.0110	0.5483	0.69
beef meat sauce	0.07	0.37	0.85
beef meat boiled	0.3	0.31	0.80
(Kitifo)Beef, raw + butter + birds eye chili	− 0.035	0.6	0.62
Yoghurt	0.3008	0.3187	0.62
Egg	0.2062	0.48	0.72
Coca-Cola	− 0.0980	0.55	0.68
Sprite	0.2006	0.32	0.85
Tea	0.19	0.25	0.89
Tela(local alcoholic beverages	0.2861	0.2417	0.85

Factor loadings ≥ 0.30 were considered significant.

### Distribution of dietary pattern scores

Participants were categorized into tertiles (T1–T3) based on dietary pattern scores. A higher proportion of males was observed in the highest tertile of the traditional dietary pattern, whereas females were more frequently represented in the lowest tertile of the mixed dietary pattern. Participants residing in rural areas were more commonly represented in the middle tertile of the traditional dietary pattern (Table 3).

Educational status showed variation across tertiles: participants with college-level education or higher were more frequently represented in the lower tertile of the traditional dietary pattern, while those with primary education were more commonly observed in the middle tertile of the mixed dietary pattern (Table 4).

**Table 4:** Distribution of dietary pattern tertile by selected socio-demographic and clinical characteristics among adults with type 2 diabetes mellitus, 2024.

Variables N = 416	Dietary patterns					
	Traditional pattern			Mixed pattern		
	T1	T2	T3	T1	T2	T3
− Sex	n (%)	n (%)	n (%)	n (%)	n (%)	n (%)
Female	57(35.4)	57(35.4)	47(29.2)	63(39.1)	47(29.2)	51(31.7)
Male	80(31.9)	80(31.9)	91(36.3)	74(29.5)	90(35.9)	87(34.7)
Age						
27–34	12(25.5)	19(40.4)	16(34)	14(29.8)	17(36.2)	16(34)
35–44	53(34.6)	50(32.7)	50(32.7)	48(31.4)	53(34.6)	52(34)
45–54	29(28.7)	36(35.6)	36(35.6)	36(35.6)	31(30.7)	34(33.7)
55–82	43(38.7)	32(28.8)	36(32.4)	39(35.1)	36(32.4)	36(32.4)
Residence						
Urban	65(34.6)	56(29.8)	67(35.6)	61(32.4)	58(30.9)	69(36.7)
Rural	72(32.1)	81(36.2)	71(31.7)	76(33.9)	79(35.3)	69(30.8)
Educational status						
Primary	36(35.3)	31(30.4)	35(34.3)	28(27.5)	38(37.3)	36(35.3)
High school	37(27.4)	54(40)	44(32.6)	49(36.3)	46(34.1)	40(29.6)
College and above	38(38)	27(27)	35(35)	32(32)	34(34)	34(34)
No education	26(34.7)	25(33.3)	24(32)	28(37.3)	19(25.3)	28(37.3)
Wealth index						
Poor	41(29.9)	42(30.7)	54(39.4)	42(30.7)	55(40.1)	40(29.2)
Middle	46(33.6)	45(32.8)	46(33.6)	47(34.3)	38(27.7)	52(38)
Rich	50(36.8)	49(36)	37(27.2)	46(33.8)	44(32.4)	46(33.8)
Other comorbidity diseases						
Yes	46(30.7)	41(27.3)	63(42)	42(28)	50(33.3)	58(38.7)
No	91(34.7)	96(36.6)	75(28.4)	95(36.3)	87(33.2)	80(30.5)
DM association member						
Yes	67(30.7)	80(36.7)	71(32.6)	59(30.4)	61(31.4)	74(38.1)
No	67(30.7)	80(36.7)	71(32.6)	78(35.8)	76(34.9)	64(29.4)
Doctor advice						
Yes	77(33.3)	79(34.2)	75(32.5)	70(30.3)	75(32.5)	86(37.2)
No	60(33.1)	58(32.0)	63(34.8)	67(37)	62(34.3)	52(28.7)
Physical activity						
Yes	70(33.2)	74(35.1)	67(31.8)	66(31.3)	69(32.7	76(36)
No	67(33.3)	63(31.3)	71(35.3)	71(35.3)	68(33.8)	62(30.8)
Knowledge on recommended diet						
High level	57(37.5)	40(26.3)	55(36.2)	15(45.5)	11(33.3)	7(21.2)
Low level	80(30.8)	97(37.3)	83(31.9)	35(37.2)	12(25.5)	7(14.9)

### Factors associated with dietary patterns

Variables with a p-value < 0.25 in bivariable linear regression were entered into multivariable linear regression models to identify independent factors associated with each dietary pattern.

#### Traditional dietary pattern

In the multivariable model, the presence of comorbid conditions was inversely associated with adherence to the traditional dietary pattern (adjusted β = − 0.45; 95% CI − 0.77, − 0.13; p < 0.01). Older age was positively associated with the traditional dietary pattern among participants aged 45–54 years (adjusted β = 0.52; 95% CI 0.01, 1.03; p = 0.04) and 55–82 years (adjusted β = 0.56; 95% CI 0.06, 1.08; p = 0.03) (Table 5).

**Table 5:** The detailed results of the regression analysis are presented in Table 5.

Variables N = 416	Traditional pattern	
	Crudeβ (95% CI)	Adjusted β (95% CI)
Sex	− 0.078(− 0.27,0.12)	0.09(− 0.23,0.4)
Residence	0.048(− 0.26,0.36)	0.037(− 0.27,0.35)
Age		
35–44	− 0.19(− 0.52,0.13)	0.02(− 0.25,0.7)
45–54	− 0.069(− 0.4,0.27)	0.5(0.010,1.028)
55–82	− 0.11(− 0.45, 0.23)	0.56(0.058,1.075)
Marital status		
Married	− 0.06(− 0.29,0.167)	− 0.004(− 0.37,0.36)
Widowed	− 0.17(− 0.46,0.10)	− 0.08(− 0.57,0.4)
Divorced	− 0.2(− 0.37,0.33)	− 0.24(− 79,0.3)
Did you have other comorbidity diseases (no)	0.2(0.038,0.438)	− 0.45(− 0.77,.− 13)
Physical exercise(no)	0.057(− 0.136,0.25)	0.18(− 0.12,0.49)
Are you a member of diabetic associations? (no)	0.4(− 0.14,0.23)	− 0.2(− 0.56,0.06)
Knowledge level	− 0.3(− 0.6,0.02)	− 0.30(− 0.09,0.8)
Wealth index		
Middle	0.16(− 0.21,0.53)	0.18(− 0.18,0.55)
Rich	− 0.126(− 0.36,0.11)	− 0.24(− 0.6,0.13)

Variables with p < 0.25 in bivariable analysis were entered into the multivariable linear regression model. Statistical significance declared at p < 0.05.

#### Mixed dietary pattern

Occupational status was significantly associated with the mixed dietary pattern. Being a daily laborer was positively associated with higher adherence to the mixed dietary pattern (adjusted β = 0.10; 95% CI 0.03, 0.18; p = 0.01). No significant associations were observed with sex, residence, educational status, comorbidity status, or dietary knowledge after adjustment.

Factor loadings of food items for identified dietary patterns among adults with type 2 diabetes mellitus at Wolaita Sodo University Comprehensive Specialized Hospital, Southern Ethiopia, 2024 (Table 6).

**Table 6:** Presents the multivariable regression results for the mixed dietary pattern.

Variables N = 416	Mixed pattern	
	Crude β (95% CI)	Adjusted β (95% CI)
Sex	− 0.3(− 76,0.07)	− 0.2(− 6,0.2)
Residence	− 0.17(− 0.37,0.01)	− 0.138(− 0.59,0.3)
Age	0.035(− 0.29,0.36)	− 0.2(− 0.9,0.49)
35–44	− 0.07(− 0.42,0.27)	− 0.5(− 1.2,0.2)
45–54	− 0.07(.− 41,0.265)	− 0.35(− 1.0,0.38)
55–82	− 0.07(.− 41,0.265)	− 0.35(− 1.0,0.38)
Occupations	0.6(− 0.15,1.07)	1.2(0.3,0.2)
Daily laborer	− 0.13(− 0.43,0.17)	0.09(− 0.57,0.77)
Private organization	− 0.16(− 0.46,0.135)	− 0.2(− 0.8 , 0.4)
Farmer	− 0.02(− 0.35,0.3)	0.5(− 0.18,0.1.27)
Merchant	0.29(− 0.23,0.8)	− 0.1(− 0.1,0.9)
Student	0.2(− 0.20,0.69)	0.58(− 0.3,1.4)
House wife	0.2(− 0.20,0.69)	0.58(− 0.3,1.4)
Educational status	0.07(− 0.14,0.3)	− 0.26(− 0.89,0.37)
Primary	− 0.1(− 0.38,0.12)	0.08(− 0.56,0.7)
high school	− 0.01(− 0.29,0.26)	− 0.3(− 0.97,0.3)
College and above	− 0.01(− 0.29,0.26)	− 0.3(− 0.97,0.3)
Did you have other comorbidity diseases(no)	− 0.19(− 0.39,0.0089)	− 0.3(− 0.75,0.1)
Do you have Family history of diabetes(yes)	− 0.19(− 0.39,0.0089)	− 0.3(− 0.75,0.1)
Have you made a Complete change of your dietary habit when you know you are diabetic(no)	0.02(− 0.17,0.2)	− 0.27(− 0.69,0.15)
Does your doctor give you advice about dietary pattern to be followed (no)	− 0.16(− 0.35,0.03)	− 0.3(− 0.7,0.12)
Knowledge level(low level)	− 0.008(− 0.2,0.19)	− 0.37(− 0.09,0.8)

Adjusted β coefficients are from multivariable linear regression analysis; p < 0.05 considered statistically significant.

Descriptions of dietary patterns among patients who are on follow up in Wolaita Sodo comprehensive specialized hospital 2024.

Majority of female participant resided in lowest tertile of the mixed dietary pattern while majority of male participant reside in highest tertile of traditional dietary pattern.

Participants with the highest tertile of the mixed dietary patterns tended to reside equally in urban and rural area, while those with the middle tertile of traditional dietary pattern resided more in the rural area.

Majority Participants, who completed primary education, reside on middle tertile of mixed dietary pattern while majority of participants, who completed High school education, resided in meddle tertile of traditional dietary pattern and participants who completed college and above education reside in lowest tertile of traditional dietary pattern. Participants, who have no education resided equally in both lowest and highest tertile of mixed dietary patterns (Table 4).

## Discussion

This study explored dietary patterns and associated factors among adults with type 2 diabetes mellitus (T2DM) attending Wolaita Sodo University Comprehensive Specialized Hospital. Using principal component analysis (PCA), two major dietary patterns—traditional and mixed—were identified, together explaining 20.7% of the total variance in food intake.

### Dietary patterns among type 2 diabetes patients

The traditional dietary pattern was characterized by consumption of bread, root vegetables, legumes, false banana–based foods, and local beverages such as tela. This pattern aligns with previous studies from Ethiopia and sub-Saharan Africa, where staple, plant-based foods remain predominant in adults’ diets^16^. Globally, similar plant-based patterns have been associated with better glycemic control and reduced cardiovascular risk^18,19^.

The mixed dietary pattern, which included raw and boiled meat, eggs, dairy products, sugar-sweetened beverages, and refined carbohydrates, resembles “Westernized” or diversified dietary patterns observed in urban populations worldwide^20,21^ . This reflects dietary transition due to urbanization, increased food availability, and exposure to processed foods. Similar findings have been reported among patients with T2DM in other African countries, including Kenya^14^ and urban Ethiopia^22^.

### Factors associated with dietary patterns

In this study, age was positively associated with adherence to the traditional dietary pattern, particularly among participants aged 45–82 years. This finding is consistent with global and regional evidence showing older adults often maintain culturally ingrained eating habits, whereas younger individuals tend to adopt more Westernized diets^9,23^.

Participants with other comorbidities were less likely to follow the traditional dietary pattern, suggesting that health conditions may prompt dietary modifications away from staple foods^21,24^.

For the mixed dietary pattern, occupational status was a significant factor. Daily laborers were more likely to adhere to this pattern, possibly due to the need for high-energy, convenient foods and limited time for meal preparation. This aligns with previous studies linking informal employment and high-energy food consumption among adults with diabetes in Ethiopia and other low- and middle-income countries^25,26^.

Interestingly, dietary knowledge was not significantly associated with either dietary pattern after adjustment, indicating that awareness alone may not translate into practice due to economic constraints, cultural preferences, and limited access to diabetes-specific nutrition counseling. Similar patterns have been observed in Ethiopia^14,27^.

### Comparison with local studies

The current findings confirm previous Ethiopian studies showing mixed adherence to dietary recommendations among adults with T2DM. For instance, studies from Addis Ababa and Jimma reported that patients often combine traditional staples with high-fat or sugar-rich foods, similar to the mixed pattern identified in this study^16,24^. This highlights persistent gaps in dietary counseling and behavioral adoption of recommended diets.

### Implications for practice

The coexistence of traditional and mixed dietary patterns underscores the need for context-specific nutrition interventions. Encouraging the consumption of plant-based traditional foods while moderating animal-source foods, added sugars, and processed products may improve glycemic control. Targeted interventions for daily laborers and other at-risk occupational groups are warranted. Integrating dietitians into chronic care, strengthening education programs, and promoting affordable local foods may enhance adherence to healthier dietary patterns^2,28^.

### Strengths and limitations

Strengths of this study include the use of PCA to identify dietary patterns and a relatively large, representative sample. Limitations include the cross-sectional design, which prevents causal inference, and reliance on self-reported dietary intake, which may be subject to recall bias. Despite these limitations, the findings provide valuable insight into dietary behaviors among T2DM patients in a resource-limited setting.

## Conclusion

This study identified two major dietary patterns among adults with type 2 diabetes mellitus in Wolaita Sodo, Southern Ethiopia: a traditional pattern and a mixed pattern, together explaining 20.7% of the total variance in dietary intake. The traditional pattern was positively associated with older age and inversely associated with comorbid conditions, while the mixed pattern was more common among daily laborers.

These findings highlight that dietary behavior among T2DM patients is influenced by socio demographic, occupational, and health factors, and not solely by knowledge. Nutrition interventions should therefore be context-specific, culturally appropriate, and targeted to high-risk groups. Encouraging the maintenance of beneficial traditional foods while moderating sugar and animal-source food intake may improve glycemic control and overall health outcomes in this population.

## Data Availability

Data and materials are available on reasonable request. Confidentiality and security of data and materials were insured through all stages of the study. The datasets generated and analyzed during the current study are available from the corresponding author upon reasonable request.

## Abbreviations

AFR: Africa region
DALYS: Disability adjusted life years
DDS: Dietary diversity score
DP: Dietary pattern
DIP: Diabetes in pregnancy
FFQ: Food frequency questioner
FIGO: International federation of gynecology and obstetrics
GI: Glycemic index
GLV: Green leafy vegetables
GDM: Gestational diabetes melts
HIP: Hyperglycemia in pregnancy
IDF: International diabetes federation
NCD: Non-communicable disease
NIDD: Non-Insulin dependent diabetes mellitus
PCA: Principal component analysis
T2D: Type 2 DM
UAE: United Arab Emirates
USA: United States of America
WHO: World Health Organization
